# Legislative Support of Quackery

**Published:** 1852-12

**Authors:** 


*Legislative Support of Quackery.*—The legislature of Georgia, at its last session, appropriated five thousand dollars to the Thomsonian, or Botanic School, located at Macon, in that state. Georgia, we believe, by this act is entitled to the *distinction* of being the first to contribute to the support of quackery from the public treasury. It is to be hoped that it will long enjoy a solitary distinction in this respect.

